# A dual-angle optoelectronic flow injection platform for ferrous ion (Fe^2+^) determination using blue LED excitation and solar cell detection

**DOI:** 10.1039/d5ra08467a

**Published:** 2026-01-14

**Authors:** Ghufran K. Allawi, N. S. Turkey

**Affiliations:** a University of Baghdad, College of Science, Department of Chemistry Baghdad Iraq ghufran.k.allawi@fosci.uoqasim.edu.iq nagham.turkey@sc.uobaghdad.edu.iq; b Al-Qasim Green University, College of Food Science, Department of Food Science and Technology Babylon Iraq

## Abstract

Herein, a new optoelectronic flow injection method was proposed for the determination of ferrous ions (Fe^2+^) based on thiocyanate complexation to form a deep-red FeSCN^2+^ complex. The system used in this method included a home-built photometric platform, a dual-angle photonic irradiation spectrometer (DAPIS), with eight high-power blue LEDs (1.5 W each) as structured light sources and two photovoltaic solar cells as detectors. The LEDs were housed inside a brass enclosure of 2 mm inner diameter placed around a 100 mm flow quartz tube, allowing dual-angle irradiation (0–90° and 0–180° interaction by internal optical reflection effects). This geometry provided high photon utilization, reducing optical loss and improving detection sensitivity. A dual-line flow injection procedure based on the use of sodium persulfate (Na_2_S_2_O_8_) as an oxidant and thiocyanate (SCN^−^) as a chromogenic reagent is presented herein. The linear dynamic range was between 0.2 and 17 mM, which was relatively wide, with an LOD of 8 µM and a correlation coefficient (*r*) of 0.99922, demonstrating the accuracy of the method and the reusability of the system. This method was successfully extended to pharmaceutical formulations and tested for statistical validation using the paired *T*-test (*t*-cal. (−0.184) < *t*-tab. (4.303)) as well as one-way ANOVA (*F*-cal. (0.069) < *F*-tab. (5.14)); no significant difference was observed between the proposed method and the conventional spectrophotometric methods. Owing to its modular design, high sensitivity, and adaptability to colorimetric, fluorometric, or turbidimetric reactions, the developed platform holds great potential for applications in environmental monitoring and agricultural analysis.

## Abstract 2

A new optoelectronic flow injection method is proposed for the determination of ferrous ions (Fe^2+^) based on thiocyanate complexation to form a deep-red FeSCN^2+^ complex.

## Graphical abstract

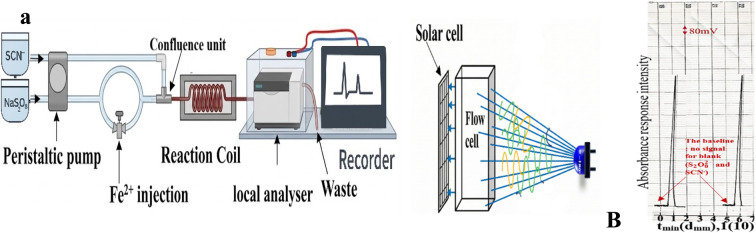

## Introduction

Iron(ii) (Fe^2+^) ion is a transition metal cation in the +2 oxidation state.^1^ It easily forms coordination complexes with donor ligands, with the primary geometry being octahedral, though it may vary based on the type of ligand and specific conditions. In general, Fe^2+^ displays high-spin configurations owing to its moderate ligand field strength, especially in aquatic and biological systems, and its facile redox interconversion with Fe^3+^ underpins both metabolic and industrial significance.^2–4^ Biologically, Fe^2+^ is indispensable for almost all organisms, serving crucial roles in oxygen transport (as the functional form of iron in haemoglobin and myoglobin), electron transfer (in cytochromes and iron-sulphur clusters that drive mitochondrial respiration and ATP synthesis), and as a cofactor for diverse enzymes such as catalase, peroxidase, and dioxygenases. Fe^2+^ deficiency is a major global health concern, frequently manifesting as iron-deficiency anaemia, which leads to impaired oxygen transport, fatigue, and hindered cognitive development.^1,5^

Several optical sensing strategies have been developed for the sensitive detection of Fe(ii) ions across environmental and biological matrices, relying mainly on spectrophotometric and colorimetric approaches. For instance, a novel spectrophotometric and optical sensor was fabricated *via* the direct immobilization of a newly synthesized chromophore, 5-amino-phenanthrolin-3-formyl salicylic acid, onto nanocellulose. Human vision can recognize the colour linked to the presence of Fe(ii) ions in human blood serum with a detection limit of 0.339 ppb.^6^ In the same manner, a dual cation-targeted colorimetric probe composed of ascorbic acid embedded in a polyazomethine matrix exhibited effective sensing capabilities for Al(iii) and Fe(ii) ions in both tap and seawater, as well as in bovine serum albumin, achieving a detection limit of 0.185 µM *via* UV-Vis and fluorescence measurements.^7^ Gold nanoparticle-based sensing systems have also been reported. The gold nanoparticles (AuNPs) were synthesized utilizing methylene blue (MB) as the capping agent and sodium borohydride as the reducing agent under aqueous conditions, which enabled the colorimetric detection of Fe(ii) and Cr(iii) ions with a detection limit of 11.21 mM.^8^ In addition, classical spectrophotometric analysis employing 1,10-phenanthroline, supported by the redox conversion of Fe(iii) into Fe(ii) *via* ascorbic acid or Ce(iv), has been integrated into lab-in-syringe and sequential injection analysis systems, allowing the reliable determination of iron species in groundwater and wastewater with a detection limit of 0.02 mg L^−1^.^9^

Additionally, flow injection analysis (FIA) is one of the most disruptive innovations in analytical chemistry, providing a continuous, automated, and highly reproducible method for quantitative measurements of several analytes. Since its introduction in the 1970s, FIA has become a versatile and reliable approach for determining pharmaceutical chemicals, transition metal ions, and ionic species in simple and complex matrices.^10–17^ The system works by accurately injecting a sample aliquot into a carrier stream, which mixes, reacts, and detects ions under well-defined hydrodynamic conditions. The system exhibits accuracy, reproducibility, and temporal control, and is indispensable for flow-based modern analysis. It requires microliters of reagents and samples and performs analyses in seconds or minutes. This method allows for low-cost operations and reduces waste, aligning with the principles of green analytical chemistry. The automated and computer-controlled method of FI can be used for high-throughput pharmaceutical analysis, process analytical technology (PAT), and kinetic studies. The modular platform of FIA also makes an easy interconnection with home-made reaction coils, injector valves and flow cells for special analytical requirements. They are versatile, reproducible, and analytically reliable, as they can be coupled with multiple detection modes, including photometric, fluorometric (including chemiluminescence-based), and electrochemical techniques, while also allowing effective confinement and interaction of the analyte species.^18–25^

Because of its rapidity, sensitivity, and low cost, FIA is a very popular technique for pharmaceutical quality control applications as well as environmental monitoring and clinical analysis. In this paper, a spectrophotometric FIA system is described, which was developed for accurate and rapid determination of ferrous ions (Fe^2+^) based on an earlier concept. By means of patterned optical irradiation, the instrument is equipped with eight ultra-high-power blue LEDs (1.5 W each) in a visible matrix surrounding a flow-through tubular cell (outer diameter: 4 mm; inner diameter: 2 mm). This optical arrangement provides uniform irradiation and enhances the interaction of photons from the reaction zone with the analyte. The response recorded through dual 37.8 mm-solar cell photodetectors at 0° and 180° relative to the irradiation axis quantifies transmitted and reflected light for analytical precision.

## Reagents and chemicals

Standard solutions were prepared following established analytical methods. A 0.25 M ammonium thiocyanate (NH_4_SCN) solution was prepared by accurately weighing 4.758 g of NH_4_SCN (MW = 76.13 g mol^−1^, BDH, UK), dissolving it in approximately 100 mL of deionized water and diluting it to 250 mL. Likewise, a 0.25 M sodium persulfate (Na_2_S_2_O_8_) solution (MW = 238.10 g mol^−1^, BDH, UK) was prepared by dissolving 14.88 g of the salt in 100 mL of deionized water and diluting it to 250 mL. For the preparation of a 0.1 M ammonium iron(ii) sulfate hexahydrate solution [FeSO_4_·(NH_4_)_2_SO_4_·6H_2_O], 9.8035 g of the crystalline salt (MW = 392.14 g mol^−1^, Fluka Chemie AG, Germany) was dissolved in 50 mL of deionized water, which had been acidified with 5 mL of concentrated sulfuric acid, and the solution was diluted to 250 mL with deionized water. The acidic medium stabilized Fe^2+^ ions by preventing aerial oxidation to Fe^3+^, thereby ensuring solution stability for subsequent redox applications.

In addition, 0.02 M solutions of hydrochloric acid (HCl, 37% w/w, density = 1.18 g mL^−1^, MW = 36.46 g mol^−1^), acetic acid (CH_3_COOH, 99.8% w/w, density = 1.05 g mL^−1^), nitric acid (HNO_3_, 70% w/w, density = 1.42 g mL^−1^, MW = 63.01 g mol^−1^), sulfuric acid (H_2_SO_4_, 96% w/w, density = 1.84 g mL^−1^, MW = 98.08 g mol^−1^), and phosphoric acid (H_3_PO_4_, 85% w/w, density = 1.84 g mL^−1^, MW = 97.99 g mol^−1^) were prepared by appropriate dilution of the concentrated stock reagents (BDH, UK). To obtain 500 mL of each acid solution at the desired molarity (0.02 M), accurately measured volumes of 0.835 mL, 0.573 mL, 0.634 mL, 0.555 mL, and 0.627 mL, respectively, were diluted to required volumes with deionized water. The prepared acid solutions were then standardized against a primary standard sodium carbonate solution (Na_2_CO_3_, MW = 105.99 g mol^−1^) to ensure accurate concentration measurement.

### Preparation and evaluation of ferrous ion tablet samples from market pharmaceutical products

Assessing the pharmaceutical quality of ferrous ion tablets from different pharmaceutical companies: three responding representative samples—Accord (UK), Awamedica (Iraq), and Iron Oshar pharma (Syria)—were prepared for analysis. The average weight of 20 tablets of each product was 0.450 g, 0.2886 g, and 0.723 g. For the final concentration of 50 mM, actual weights were measured from each sample (1.710 g, 1.096 g and 2.80 g). The weighed quantities were then pulverized using a mortar and pestle to ensure the homogenization of an active pharmaceutical ingredient. The resulting powdered samples were then quantitatively transferred into a 100 mL volumetric flask, and the volume was made up to mark with distilled water while stirring continuously for dissolution. The solutions were sonicated for 10 min to promote the dissolution and homogeneous dispersion of ascorbic acid. The solutions were filtered through a 0.45 µm membrane filter before other analytical methods were performed. The last preparations were kept in amber glass vials at controlled room temperature to avoid oxidative degradation until analysis.

### Apparatus

**Fig. 1:**
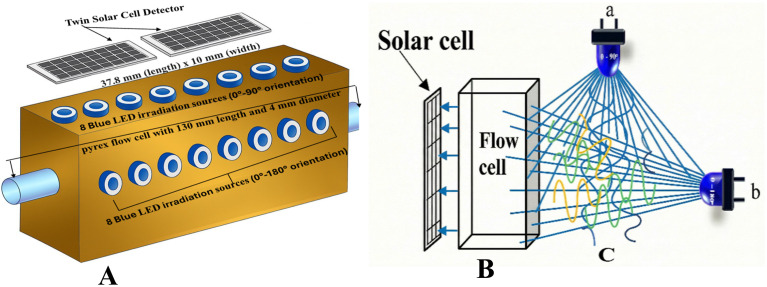
Illustrating the main components of the spectroscopic instrument along with the measurement configuration: (A) measurement cell, (B) flow cell with a solar cell as a detector and (C) illuminated area by (a) source at 0–90° and (b) source at 0–180°.

A local photometric detecting device,^14,26^ designated DAPIS, was constructed and utilized as the primary analytical unit in this investigation. The internally constructed device was affixed to a brass incubator block of 100 × 40 mm size, with two sets of eight precision-drilled lateral holes of identical dimensions, positioned at angles of 0–90° and 0–180° relative to the detection axis, as illustrated in Fig. 1. The initial assembly comprised blue light-emitting diode (LED) arrays (1.5 W, 5 mm diameter) positioned at a depth of around 14 mm, whereas the subsequent configuration is capable of bending from 0° to 180°, accompanied by a dual photovoltaic cell unit for signal acquisition. Four programmable irradiation levels separately control each LED array, allowing for the precise modulation of the photon flux (P°) to which the analyte stream is subjected by voltage variation. This configuration provides both adaptability for field position angles and customizable intensity, features typically absent in commercial photometers. The two-illumination geometry is a crucial characteristic, as it provides distinct (or concurrent) illumination of each array, facilitating spectral symmetry and self-absorption interference correction. These factors enhance signal resolution and broaden analyte detection, particularly in complex matrices.

The flow injection system was operated using a four-channel peristaltic pump (IPC-N), which allowed precise and adjustable control of all carrier and reagent flow rates. Sample introduction was performed *via* a six-port medium-pressure injection valve (model 7725i) equipped with a Teflon sample loop (1 mm id, variable length).

Signal acquisition and real-time monitoring of the optoelectronic response were performed using a potentiometric recorder (REC-10; 1–5 V range), which continuously measured the voltage output generated by the solar cell detector(s) as a function of time. The recorded signals were subsequently processed and analyzed using OriginLab software.

For comparison studies using the conventional method, a UV-visible detector (SPD-20A) coupled with the LabSolutions data acquisition software was employed for absorbance measurement and data processing.

## Methodology

**Fig. 2:**
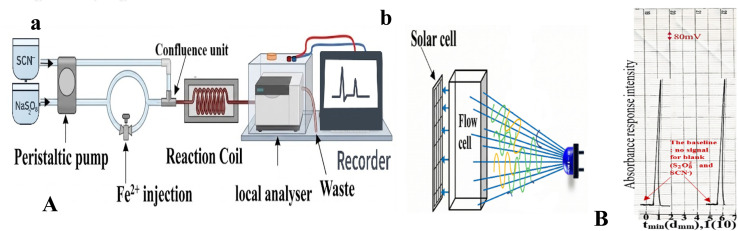
(A-a) Schematic of a dual-line flow injection analysis system for Fe^2+^ determination, showing the first line with a carrier stream for the oxidation of Fe^2+^ (after introduction through the injection valve) to Fe^3+^ and subsequent transport by sodium persulfate. The oxidized Fe^3+^ stream then merges with the thiocyanate reagent line at a Y-junction confluence, where complexation occurs to form the red-coloured FeSCN^2+^ complex. The Y-junction confluence followed by a reaction coil for complex development and the final analyser unit. (A-b) Representation of the analyser unit consisting of an LED-blue irradiation source with a single solar cell detector positioned at the 0–180° measurement angle. (B) Display of the actual profile output response, replicated twice at 13 mM for Fe^2−^, 10 mM for S_2_O_8_^2−^ and 12 mM for SCN^−^.

A dual-line flow injection system was developed for the spectrophotometric determination of ferrous ions (Fe^2+^), as shown in Fig. 2A-a. In the first line, a carrier stream of sodium persulfate (Na_2_S_2_O_8_) at a concentration of 10 mM was used to transport a 125 µL sample of ferrous ion at a flow rate of 1.5 mL min^−1^. The oxidation of Fe^2+^ to Fe^3+^ occurred within the injection valve, where sodium persulfate acted as the oxidizing agent, following the reaction:Fe^2+^ + S_2_O_8_^2−^ → Fe^3+^ + 2SO_4_^2−^

Simultaneously, in the second line, a 12 mM solution of thiocyanate ions (SCN^−^) flows at a flow rate of 1.4 mL min^−1^. The two streams merged at a mixing junction, where the freshly generated Fe^3+^ reacted with SCN^−^ to form the red-coloured ferric thiocyanate complex (FeSCN^2+^), according to the following equation:Fe^3+^ + SCN^−^ → FeSCN^2+^

The resulting FeSCN^2+^ complex was directed into a detection cell irradiated by eight blue light-emitting diodes (LEDs). The cell was equipped with twin solar cells positioned at an angle of 0–180° relative to the light sources, as illustrated in Fig. 2A-b. The optical path length was maintained at 2 mm, which directly corresponds to the internal diameter of the flow tube used as the flow cell.

In the developed dual-line flow injection system, the blank signal was defined as the baseline generated by the carrier and reagent streams in the absence of Fe^2+^ ions. The blank consisted of sodium persulfate (S_2_O_8_^2−^) in the carrier line and thiocyanate (SCN^−^) in the reagent line. Upon merging of both streams without sample injection, no FeSCN^2+^ complex was formed, resulting in a stable baseline signal. The injection of Fe^2+^ produced a measurable optoelectronic response due to complex formation. Therefore, the baseline served as the blank, and no separate blank subtraction was required, as demonstrated in Fig. 2B.

The photocurrent responses generated by the solar-cell detector(s) were recorded, and each measurement was performed in triplicate to ensure reproducibility and reliability. Absorbance-related responses were monitored using a calibrated strip-chart recorder, and all recorded data were subsequently processed using OriginLab software.

## Results and discussion

### Spectral characteristics and electronic transitions of the FeSCN^2+^ complex *versus* Fe^2+^ ions

**Fig. 3:**
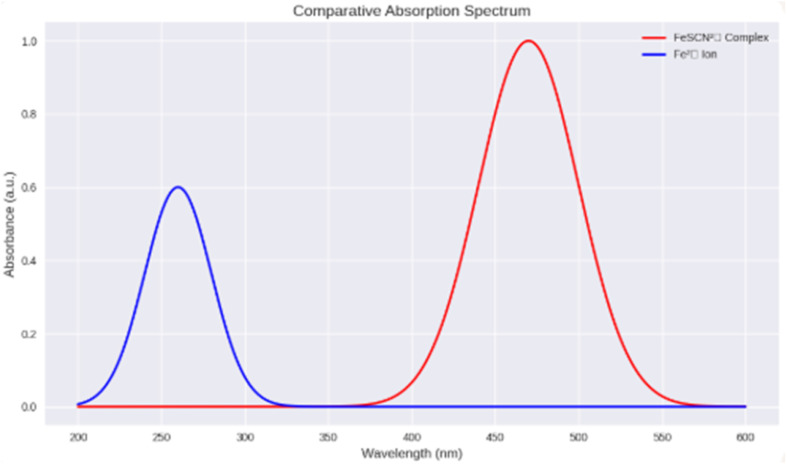
UV-Vis absorption profiles of the FeSCN^2+^ complex (*λ*_max_ = 470 nm) and free Fe^2+^ ions (*λ*_max_ ≈ 260 nm), illustrating the enhanced visible-region response of the coloured complex.

The comparative absorption spectrum reveals distinct optical behaviours between the FeSCN^2+^ complex and the free Fe^2+^ ion. The FeSCN^2+^ complex provides a pronounced absorbance peak at 470 nm, situated within the visible region, which corresponds to a ligand-to-metal charge transfer (LMCT) transition. This transition arises from the donation of electron density from the thiocyanate ligand (SCN^−^) to the vacant d-orbitals of the Fe^2+^ centre, to result in the creation of a deeply coloured red complex. The Fe^2+^ ion alone displays a weak absorbance band cantered around 260 nm (Fig. 3), attributed to intra-ligand or d–d transitions that are typically Laporte-forbidden and less intense in aqueous environments. The different spectral patterns show the significance of ligand coordination in changing the electronic structure and optical properties of transition metal ions. The LMCT band of FeSCN^2+^ makes things simpler to detect in the visible spectrum and allows optoelectronic systems that use blue-light excitation sources take accurate measurements. Simultaneously, the UV region absorbance of Fe^2+^ necessitates more sensitive instrumentation, and is less appropriate for flow-based detection platforms relying on visible light. This spectral feature is the foundation for the enhanced analytical performance that has been observed in the developed system.

### Effect of sodium persulfate concentration on FeSCN^2+^ complex formation

**Fig. 4:**
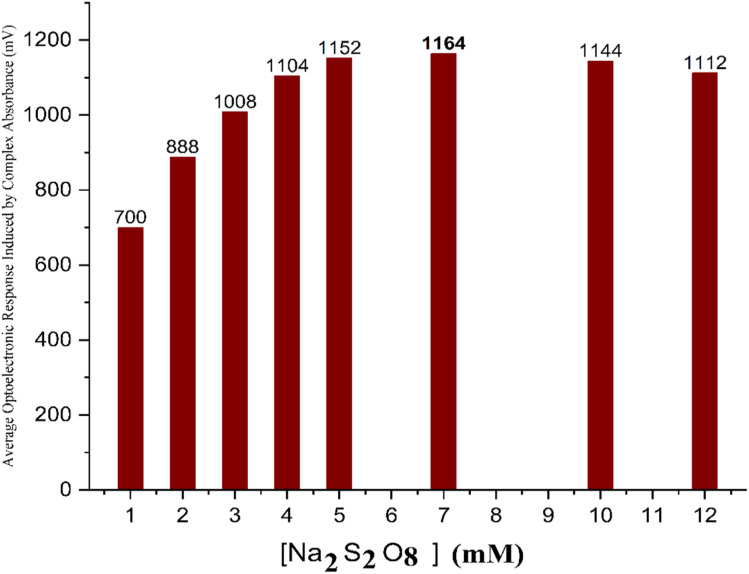
Illustration of the trend in photocurrent response as a function of sodium persulfate concentration, confirming the optimal value at 7 mM.

The influence of sodium persulfate concentration on the formation of the red-colored FeSCN^2+^ complex was systematically investigated. A series of experiments were conducted by varying the concentration of Na_2_S_2_O_8_ from 1 mM to 10 mM while maintaining constant conditions for other parameters (concentration of Fe^2+^ was 12 mM and that of SCN^−^ was 3 mM, at a flow rate of 1.25 mL min^−1^ with a sample volume of 100 µL). The findings disclosed a direct proportional relationship between the oxidant concentration and the intensity of the complex signal up to an optimal concentration of 7 mM, as illustrated in Fig. 4. This improvement is due to the increasing prevalence of oxidizer ions. This stimulates the complete oxidation of Fe^2+^ to Fe^3+^, resulting in more effective complexation, thereby enhancing effective complexation with SCN^−^ ions. This quenching effect is probably due to excessive persulfate ions causing side reactions or over-oxidation, which may interfere with the stability or formation of the FeSCN^2+^ complex. Such behaviour is consistent with oxidative suppression phenomena is reported in classical analytical chemistry.

### Effect of the thiocyanate ion concentration on the photocurrent response

**Fig. 5:**
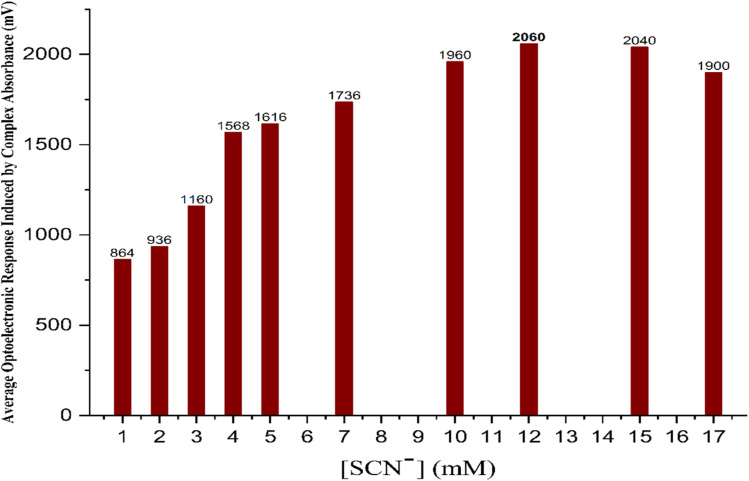
The effect of the SCN^−^ concentration on photocurrent response, confirming the optimal value at 12 mM.

The influence of the concentration of thiocyanate ions (SCN^−^) on both the formation of the FeSCN^2+^ complex and its photocurrent response was also studied under fixed experimental conditions, with the previously optimized sodium persulfate concentration adjusted to 7 mM, allowing full oxidation of Fe^2+^ (12 mM). The mean volume of SCN^−^ was between 1 and 17 mM at a constant flow rate of 1.25 mL min^−1^ and an injection volume of 100 µL under irradiation conditions. The detection section had 8 pcs of blue light-emitting diodes (LEDs) as excitation sources and two solar cells that were disposed at an angle from 0° to 180° with respect to the light-receiving direction. The optical path length was set at 2 mm to guarantee the same conditions of light interaction with the complex formed. The results revealed that there was a linear relationship between the concentration of SCN^−^ and the photocurrent response, which increased until it reached its maximum value at 12 mM SCN^−^, after which it decreased slightly. The response in the beginning is attributed to excess production of the FeSCN^2+^ complex, which has a high absorbance in the visible light, boosting up photon capture and driving the energy conversion into solar cells. The result is in accordance with the Beer–Lambert law, implying that higher absorbance and larger analytical signals can be obtained with the increase in chromophore concentration. However, when it exceeded 12 mM of SCN^−^ concentration, the photocurrent response declined. This quenching would be caused by ligand oversaturation, leading to competitive side reactions and the production of other species that do not contribute to the absorbance we are looking for. Furthermore, the presence of high SCN^−^ levels can increase solution turbidity or lead to light scattering, which are both detrimental to the transmission and absorption of the light in the sensor cell. The peak in the results given in Fig. 5 is at 12 mM SCN^−^.

### Influence of the oxidation medium on the complex formation and optoelectronic response

**Fig. 6:**
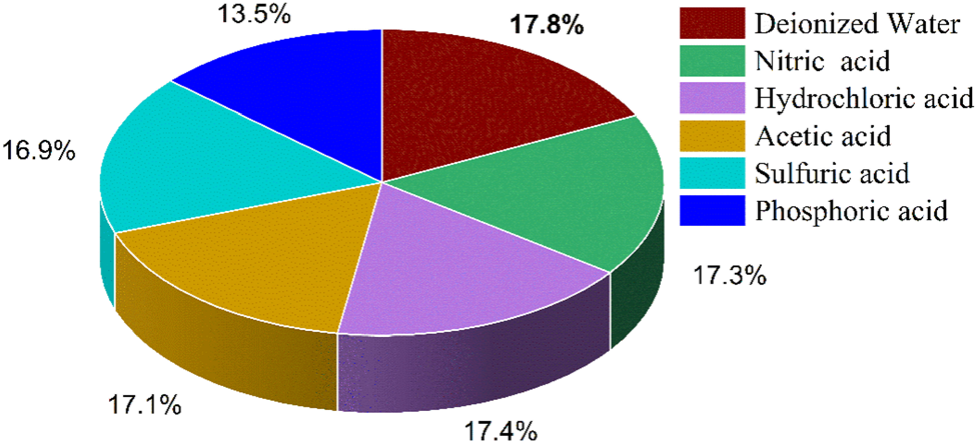
A pie chart illustrating the photocurrent response as a percentage and showing the effect of differences in oxidation medium, confirming the optimal value obtained using deionized water.

To evaluate the effect of different oxidation media on the formation of the FeSCN^2+^ complex and its corresponding optoelectronic response, a series of acidic environments including nitric, hydrochloric, acetic, sulphuric, and phosphoric acids were tested in comparison to deionized water. A ferrous ion (Fe^2+^) solution with a fixed concentration of 12 mM was prepared and diluted using each medium. The resulting solutions were injected as 100 µL samples into the flow system at a rate of 1.25 mL min^−1^ under optimal conditions as found in previous sections, and constant irradiation and detection conditions. The optoelectronic response, measured as voltage output from the solar cell detection system, was used to assess the efficiency of complex formation. Deionized water was the optimal effective medium in all investigations, as it provided the highest signal intensity and demonstrated greater complex stability and absorbance, as shown in Fig. 6. Inversely, none of the acidic solutions used improved both sensitivity and colour complex absorbance. This conclusion indicates that strong acids might perturb the coordination environment or ionic strength in some way, which hampers the best possible complexation. In both cases, results reveal that deionized water represents the best media suitable for further analytical studies on the formation and detection of the FeSCN^2+^ complex by optoelectronic transduction.

### Effect of the flow rate on the optoelectronic response

**Fig. 7:**
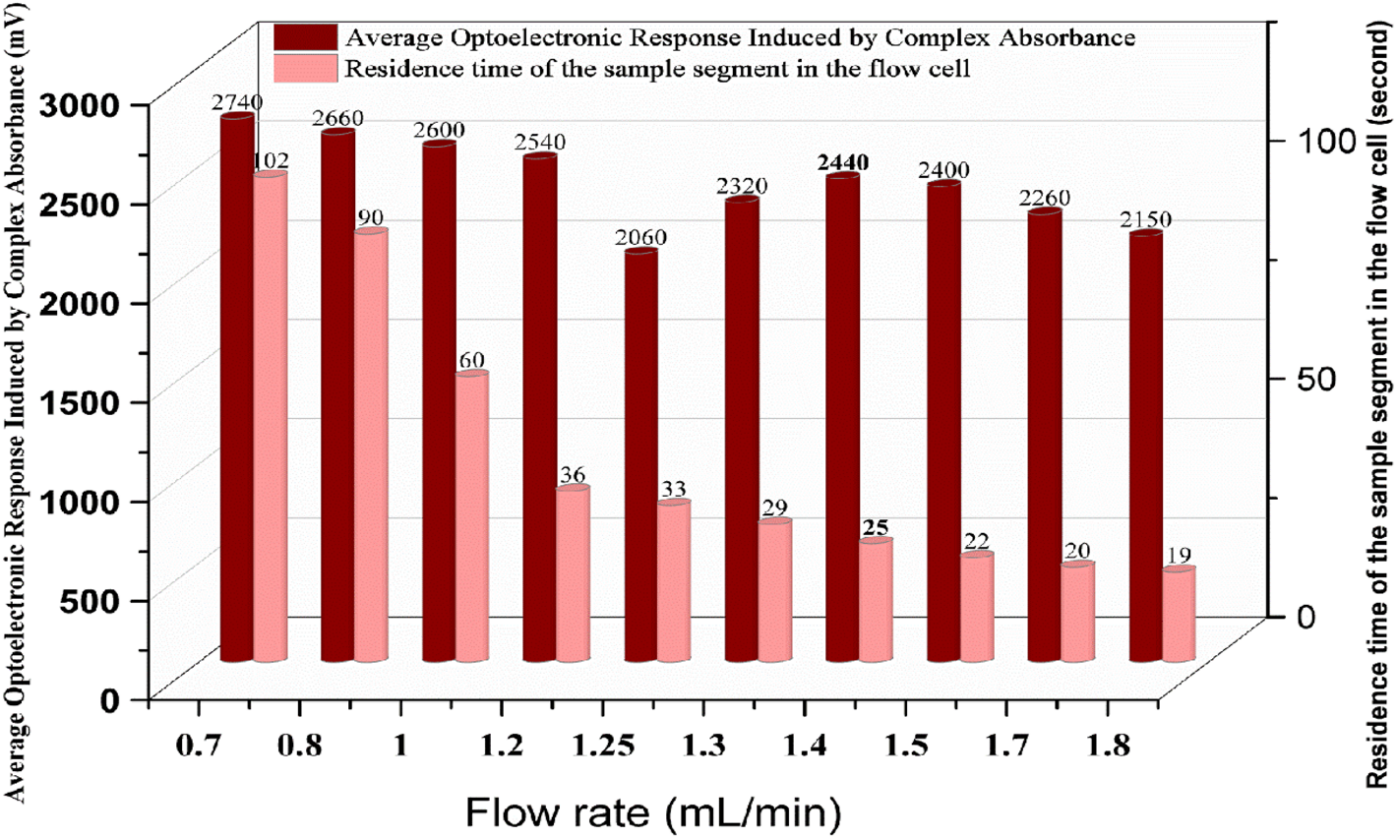
Influence of different flow rates on the optoelectronic response of the FeSCN^2+^ complex, confirming the optimal value obtained to be 1.4 mL min^−1^.

The influence of the flow rate on the optoelectronic response of the FeSCN^2+^ complex was investigated over a range of 0.7 to 1.8 mL min^−1^, using a fixed injection volume of 100 µL, and the optimal chemical parameter was archived in a previous study under constant irradiation conditions. The flow rate has a direct effect on the residence time of the sample segment (represented the baseline width of the peak) in the flow cell (detection zone), thus impacting signal stability, dispersion, and mass transfer. At lower flow rates (≤1.0 mL min^−1^), increased residence time enhances diffusional effects and axial dispersion, leading to broader peaks and reduced signal sharpness. Conversely, at higher flow rates (≥1.6 mL min^−1^), excessive dilution and insufficient interaction time between the complex and the light source result in diminished sensitivity and signal flattening. The optimal flow rate was determined to be 1.4 mL min^−1^, which yielded sufficient signal intensity with an acceptable residence time, resulting in minimal dispersion and an optimal peak height, as illustrated in Fig. 7.

### Effect of the sample volume on the signal profile

**Fig. 8:**
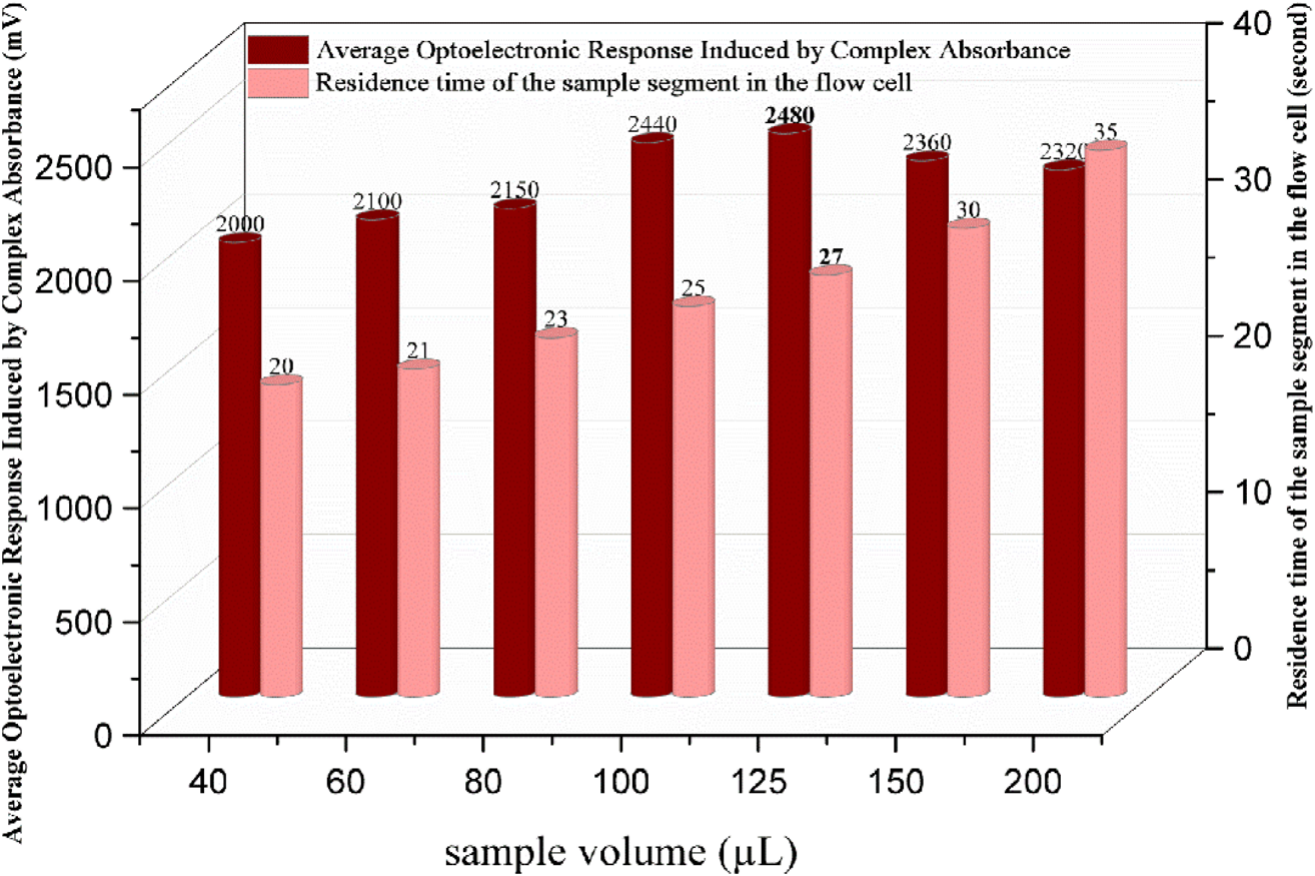
Influence of different sample volumes on the optoelectronic response of the FeSCN^2+^ complex, confirming the optimal value obtained to be 125 µL.

To evaluate the effect of sample volume on the formation and detection of the FeSCN^2+^ complex, injection volumes ranging from 40 to 200 µL were investigated under the previously optimized chemical conditions. A volume of 125 µL was selected as optimal based on the signal intensity and peak morphology, as shown in Fig. 8. Increasing the sample volume improves the formation of coloured complexes, which, in turn, enhances the absorbance and signal magnitude. On the contrary, excessive volume may result in self-absorption, internal filtering effects, and saturation of the optoelectronic detection system. Furthermore, increased sample volume increases the residence time of the sample segment in the optical path, which gives broader peaks and increases the baseline width. These are the factors that need to be carefully balanced to avoid compromising signal integrity and peak reproducibility, which are important parameters in the context of FIA for reliable quantitation and repetitive analytical performances.

### Effect of the reaction coil volume on the complex formation

**Fig. 9:**
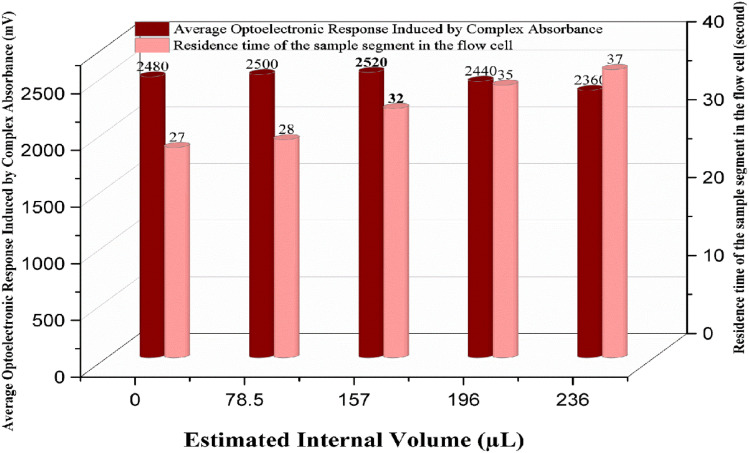
Influence of different reaction coil volumes on the optoelectronic response of the FeSCN^2+^ complex, confirming the optimal value obtained to be 15 µL.

The length of the reaction coil (*i.e.*, the distance from mixing to detection) was determined to be between 0 and 30 cm, which equated to volumes of 0–236 µL for a capillary with an internal diameter of 1 mm. We assessed its contribution to the complex formation reaction. The best choice for the length of the coil is 20 cm with an internal volume of around 157 µL, as in Fig. 9. This layout provides enough residence time for the FeSCN^2+^ complex to fully develop before reaching the detection cell and allows maximum intensity of signal. However, it is proposed that longer coils could potentially improve the reaction completion, and they are also expected to have side effects, such as greater axial dispersion and dilution of the coloured species, leading to decreased sensitivity. These effects manifest with the broadening of signal profiles, reduction in peak intensity, and widening of the baseline due to a longer residence time, which subsequently have impacts on the accuracy and performance for detection.

### Analytical and optoelectronic evaluation of the dual-angle flow injection system for the quantitative determination of ferrous ions

**Fig. 10:**
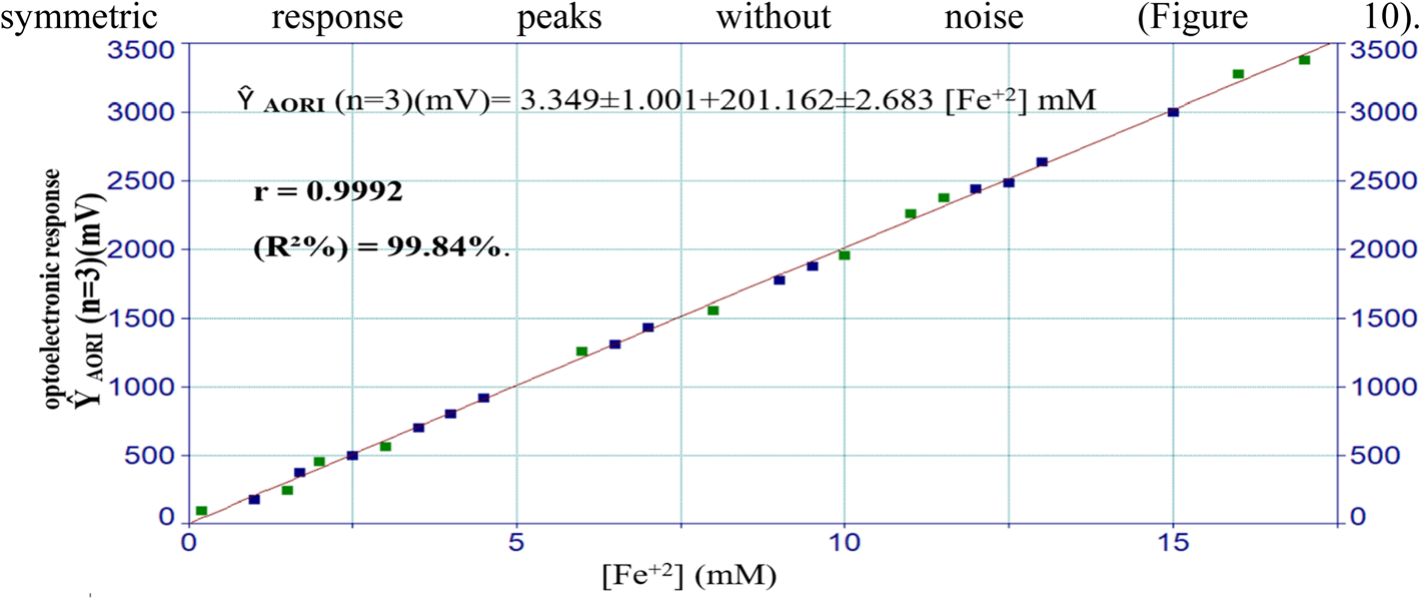
The effect of different ferrous ion concentrations on the optoelectronic response of the red complex's absorbance, as visualized through a calibration curve constructed.

The developed analysis platform features a custom-made optoelectronic flow-injection arrangement designed for the matrix-independent quantitative determination of Fe^2+^ by means of its complexation reaction with SCN^−^. The developed system, consisting of a dual-angle blue LED irradiation source, twin solar cell detectors, and a precision regulator, was optimised for achieving a highly photometrically sensitive signal, stability, and repeatability. The optimised calibration plot showed a wide linear dynamic interval from 0.2 to a maximum of 17.0 mM Fe^2+^; the *r* value was found to be 0.99922, which demonstrated the excellent proportionality between the analyte concentration and the optoelectronic signal response (Fig. 10). The corresponding regression formula is written as *Ŷ*_AORI_ (*n* = 3) (mV) = 3.349 ± 1.001 + 201.162 ± 2.683 [Fe + 2] mM, where *Ŷ*_AORI_ corresponds to the optoelectronic response induced by complex absorbance (AORI) given in millivolts (mV). *R*^2^ = 99.84%, which is close to 100%, indicating that there is a satisfactory linear relationship and low interference with random errors in the measurement range. A slight deviation from linearity was noted at concentrations exceeding 17.0 mM. This phenomenon is caused by optical saturation and inner-filtering effects, common at high chromophore concentrations when photon absorption becomes self-limited. However, even at these high concentrations, the signal was stable, reproducible, and statistically predictable. When the lowest concentrations in the calibration curves were successively diluted, the test results indicated that the system had high analytical sensitivity with an LOD of 8 µM. Intraday repeatability was evaluated by analyzing two concentration levels within the calibration range: 3.5 mM (mid-range) and 13.0 mM (upper-range). Each concentration was measured five times consecutively under identical operating conditions within the same day, and the repeatability was assessed based on these replicate measurements (*n* = 5). The RSD of three injections of each analyte was 0.86% and 0.94%, respectively, which indicated very good reproducibility and instrumental precision in the proposed method. Both the low level of signal drift and the constant peak height observed in all the replicates indicated that the irradiation–detection system was stable and that the dual-LED matrix provided a homogeneous photon flux. Additionally, the twin photocell detectors were arranged in a 180° angular configuration, which improved the optical capture efficiency and minimised alignment or scattering errors compared to configurations with sharp band symmetric response peaks without noise (Fig. 10).

This method was then validated against a typical spectrophotometric assay using a Shimadzu UV-1800 UV-Vis Spectrophotometer (Shimadzu Corporation, Tokyo, Japan). The introduction and optimization of complementary reagents involved in the complexation reaction were carried out first. After optimization, a standard calibration curve was prepared, and the absorbance was measured at a fixed wavelength of 470 nm.^27^ The classical approach presented a narrower linear dynamic range of 0.2–10 mM, as indicated by the regression equation *Ŷ*_i_ (*n* = 3) = 0.0108 ± 0.002 + 0.1980 ± 060 [Fe^2+^] mM, with a correlation coefficient (*r*) of 0.9985 and an *R*^2^ value of 71%, while the limit of detection (LOD) was greater at 50 µM than the newly developed optoelectronic system. The low performance is due to a fixed optical path length and a small light throughput characterised by a wide spectral bandwidth, all of which hamper the sensitivity and linearity. An RSD value < 0.5% was obtained considering the repeatability and at concentration levels of 3.5 mM and 6.5 mM, whereas acceptable precision was observed for higher concentrations, though signal saturation and baseline drift limited the sensitivity to some extent. In contrast, the present optoelectronic system exhibited high precision and satisfactory repeatability in a wide range of concentrations, even at higher levels such as 13 mM. Its wide linear range, low detection limits, and steady signal response all indicate its analytical performance. These features—sensitivity, linear dynamic range and structural flexibility—place the design platform as a promising alternative for the flow-based quantification of transition metal complexes.

### Comparative application of flow injection and UV-visible spectrophotometric methods for the determination of ferrous sulphate in commercial pharmaceutical formulations

The application and analytical utility of the proposed optoelectronic responses induced by the complex absorbance (AORI)-based flow injection system are explored through comparative studies of three pharmaceutical products, which claim to contain 200 mg of ferrous sulphate each, manufactured by Iron Oshar (Syria), Awamedica (Iraq) and Accord (UK). For each product, an aliquot volume of 0.2 mL of the prepared sample solution was transferred into a 10 mL volumetric flask and subjected to successive standard addition protocol from a stock solution containing Fe^2+^ ions at a concentration level of 50 mM in particular volumes: 0.0 mL, 0.2 mL, 0.4 mL, and 0.6 mL 0.8 mL, which correspond to relative concentrations of 0.0 mM, 1.0 mM, and 2.0 mM, 3 mM and 4 mM respectively. The unspiked sample was considered the matrix blank. The standard addition method (SAM) was applied for the determination, using both the developed FIA-AORI system and a conventional UV-visible spectrophotometric procedure^27^ at 470 nm under identical reagent conditions. The results of the analyses are presented in Table 1 shows a very good agreement between the two methods. The added ferrous sulfate ranged from 194.805 to 203.012 mg, with recovery values of 97.40–101.50%, indicating high accuracy. The narrow confidence interval and low standard deviation from triplicate measurements further confirm the reliability of the results.

**Table 1:** Analytical evaluation of commercial ferrous sulfate samples with reliability indicators^a^

Commercial pharmaceutical formulations	Nominal content (mg)	FI results (mg ± CI)	UV results (mg ± CI)	Recovery (%) FI, UV	*T*-test results	*F*-test result (ANOVA)
Iron Oshar (Syria)	200	194.805 ± 4.805	191.304 ± 5.832	97.40%, 95.65%	*t*_cal_ (−0.184) < *t*_tab_ (4.303)	*F* < *F*_a_, 0.069 < 5.14
Accord (UK)	200	197.022 ± 2.977	196.079 ± 3.987	98.52%, 98.04%		
Awamedica (Iraq)	200	203.012 ± 2.982	209.03 ± 4.982	101.50%, 104.52%		

a *δ*_*n*−1_: standard deviation based on replicate measurements (*n* = 3). *T*-test and ANOVA performed at *α* = 0.05; critical values from standard statistical tables. CI: confidence intervals = 
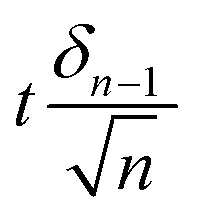
 calculated at 95% confidence level based on replicate measurements (*n* = 3). Statistical tests performed at *α* = 0.05.

## Conclusion

The in-house fabricating optoelectronic flow-based local system provides a significant progression for chemical quantitation through innovative structural and optical designs. Its performance is based on the use of 8 high-intensity blue LED sources, located to irradiate a quartz flow tube that is 100 mm long with 2 mm diameter. This arrangement provides homogeneous and effective excitation of the passing sample and allows for precise measurement over the complete working range from low to high concentrations. A significant increase in sensitivity results from the internal reflection of light at the smooth walls of a quartz tube. These reflections mimic fibre-optic action, providing an effective enhancement of the optical path length and irradiance in the flow stream. This mechanism is useful especially for the analysis of high-stained-intensity samples to minimize signal attenuations and disturbing optical noises, which can maintain a clear peak definition and S/N. The detector system FaPD is equipped with photovoltaic solar cells as detectors, aligned at any angle from 0° to 180° (or from 0° to 90°) with respect to the flow axis. The use of two blue LED irradiation arrays positioned at 0–180° and 0–90° was intentionally implemented to demonstrate the versatility of the home-built optoelectronic platform. The 0–180° configuration is particularly suited for absorbance- or turbidity-based measurements of coloured or turbid reaction products, while the 0–90° configuration enables off-axis excitation appropriate for fluorescence emission or light-scattering detection. This dual-angle architecture highlights the adaptability of the system for different optical transduction modes, depending on the nature of the chemical reaction involved. It is this versatility that ensures the suitability of the system for many chemical species, as long as a chromogenic, fluorogenic, or turbidity-inducing reaction can be used. The system exhibited satisfactory accuracy and reliability, with recovery levels at 97.403–101.506% based on an analytical validation of the system. High repeatability and low relative standard deviation at different concentration levels of the platform well confirmed its stability. Such characteristics render it particularly promising as an analytical tool for the abovementioned purposes; it can be applied for medical diagnosis, pollutant detection, and agricultural analysis, where distribution and resistance to liquid flow through porous systems are important factors in portable instruments necessitating quick, sensitive, and low-cost detection. In conclusion, the implementation of targeted excitation, improved light-sample coupling, and flexible detection geometry are the key reasons that the system developed here surpasses conventional spectrophotometric or turbidimetric techniques. The modular architecture and optical efficiency render it suitable for the quantification of various chemical analytes in flow-based analytical methods.

## Author contributions

Turkey N. S.: conceptualization, data curation, formal analysis, funding acquisition, investigation methodology project, administration, supervision. Ghufran K. Allawi: software, validation visualization, writing – original draft, resources, writing – review & editing.

## Conflicts of interest

There are no conflicts to declare.

## Data Availability

The data supporting this article are all included as part of the article.
